# Double localization of a non-anastomotic pseudoaneurysm after an axillofemoral bypass: a case report and review of the literature

**DOI:** 10.1186/s13256-016-1149-3

**Published:** 2017-01-04

**Authors:** Badr Bensaid, Tarek Bakkali, Youssef Tijani, Samir Elkhalloufi, Brahim Lekehal, Yassir Sefiani, Abess El Mesnaoui, Younes Bensaid

**Affiliations:** Vascular Surgery Department, Ibn Sina Hospital, Mohamed V University, NAHDA Avenue, Box 45, App 21, Rabat, Morocco

**Keywords:** Non-anastomotic pseudoaneurysm, Axillofemoral bypass, Ringed graft, Interposition graft, Case report

## Abstract

**Background:**

A traumatic non-anastomotic pseudoaneurysm is a rare complication of an axillofemoral bypass graft. Fewer than 20 cases have been reported in the literature. Our case is unusual in that we report a double localization of this complication.

**Case presentation:**

We report the case of a 60-year-old Arabic male patient who was diagnosed with two hematomas in the trajectory of his axillofemoral bypass secondary to a traumatism. The diagnosis of a non-anastomotic pseudoaneurysm was retained considering the results of a computed tomography angiography scan, which showed the double localization of the pseudoaneurysm. Surgical management consisted of flattening the pseudoaneurysm along with the interposition of a prosthetic segment. There were no postoperative complications and our patient was well 3 years after discharge.

**Conclusions:**

Non-anastomotic pseudoaneurysm is a rarely described complication of a axillofemoral bypass graft. To the best of our knowledge, a double localization has not been described in the literature before. Minimally invasive techniques as a treatment option are being widely used as an alternative to open repair.

## Background

In 1963 Blaisdell and Hall were the first to describe an axillofemoral bypass graft (AFBG); it has since become one of the commonly used surgical techniques. It is routinely used for lower extremity occlusive disease in selected situations. Its major indications are a hostile abdomen, multiple previous abdominal surgeries, a prohibitive risk from general anesthesia, and severely sick patients [1].

The most commonly known complications of this technique are thrombosis and infection. Other complications, such as postoperative axillary anastomosis disruption, have also been reported. According to some retrospective studies, postoperative axillary anastomosis disruption can occur in 5% of the cases [2], especially in the first few weeks after surgery [3]. Non-anastomotic pseudoaneurysm (PSA) is considered a rare complication of AFBG. Few cases have been described in the literature [4–6].

Our case report not only describes a traumatic PSA at the mid-shape of an AFBG but also adds to the literature an unusual case with a double localization.

## Case presentation

A 60-year-old Arabic male patient presented to our emergency department with pulsating masses in his right torso 5 days after a traumatism secondary to a cart accident. A review of his medical chart revealed that he had undergone an AFBG about 2 months earlier for lower limb revascularization. The indication for the AFBG was an occlusion in his primitive iliac artery, diagnosed by Doppler ultrasonography and computed tomography (CT) angiography. At that time, AFBG was chosen as the treatment of choice because of our patient’s low ventricular ejection fraction (35%) in addition to several comorbidities, including diabetes (type 2 diabetes mellitus treated with insulin) and high blood pressure (for which he was being treated), and his long history of tobacco use (active smoker for about 40 years). Revascularization was performed using an 8-mm, reinforced, thin-walled, fluorinated ethylene-propylene-ringed, expanded polytetrafluoroethylene (ePTFE) graft.

**Fig. 1:**
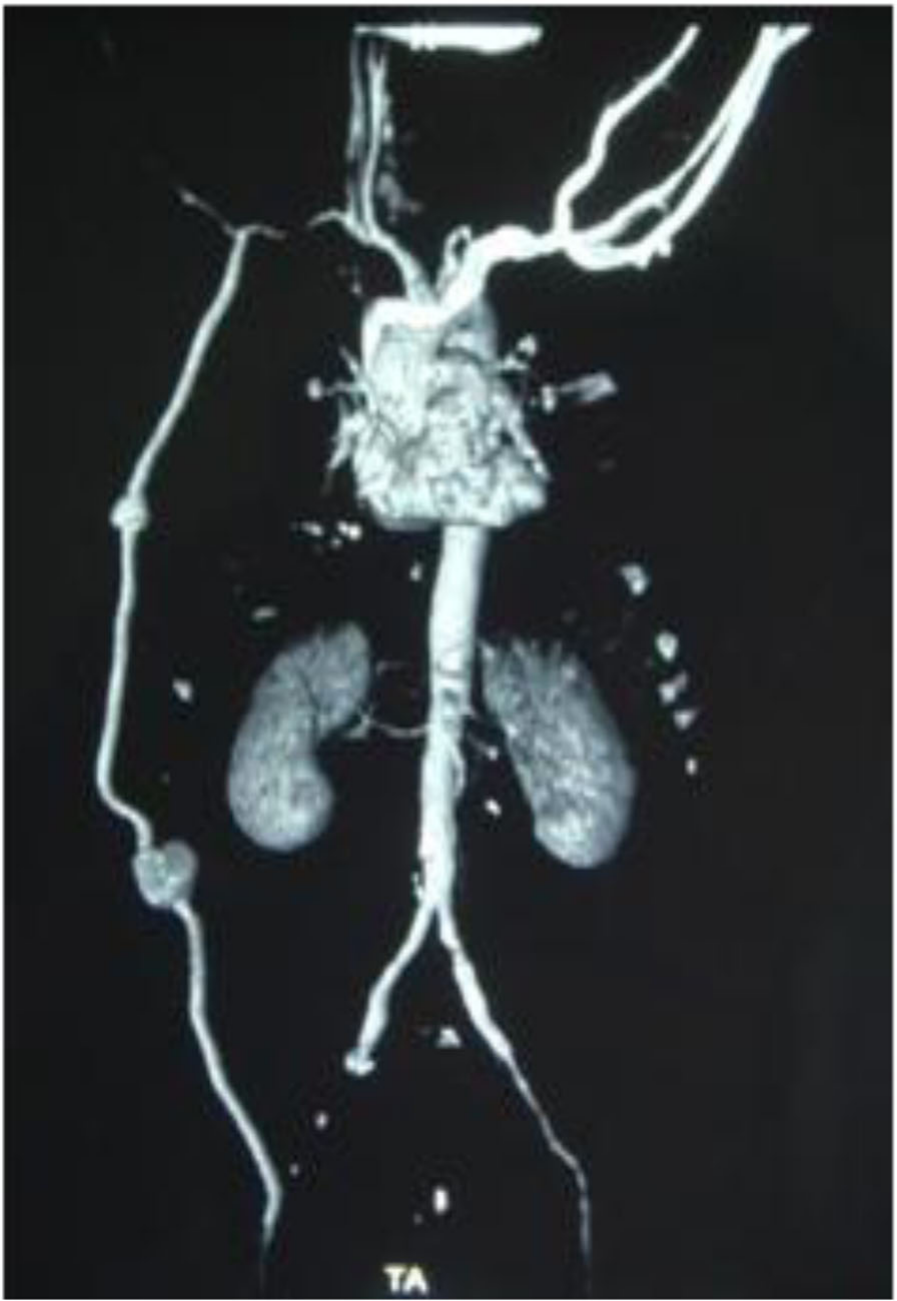
Three-dimensional vessel reconstruction from biplane angiogram: double localization of non-anastomotic pseudoaneurysms

**Fig. 2:**
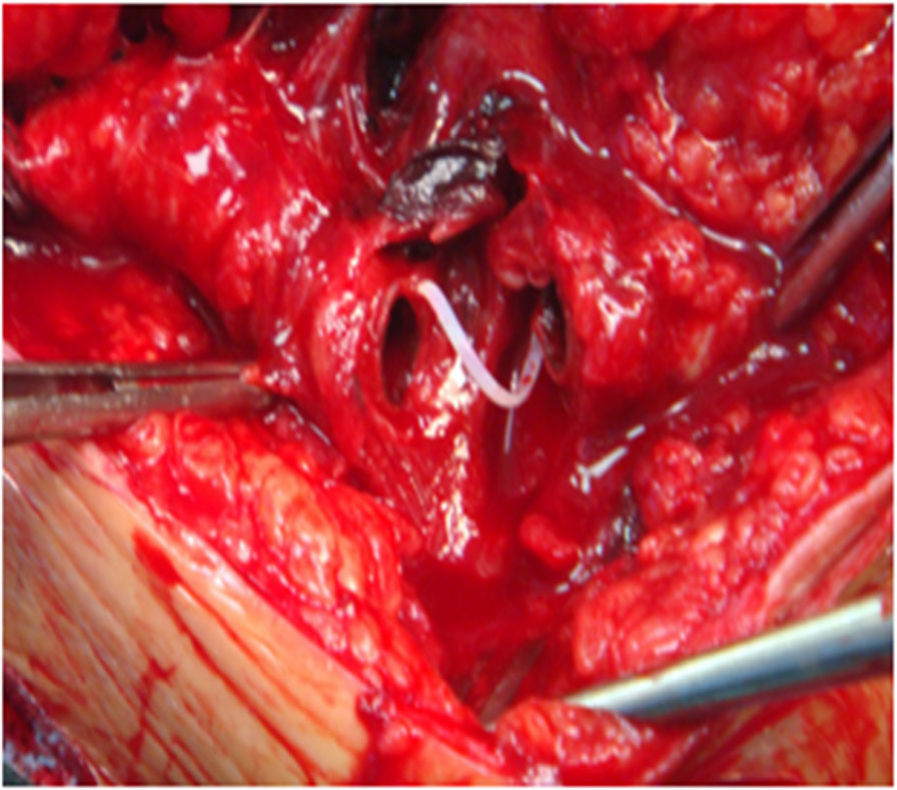
Intraoperative view of the pseudoaneurysm

**Fig. 3:**
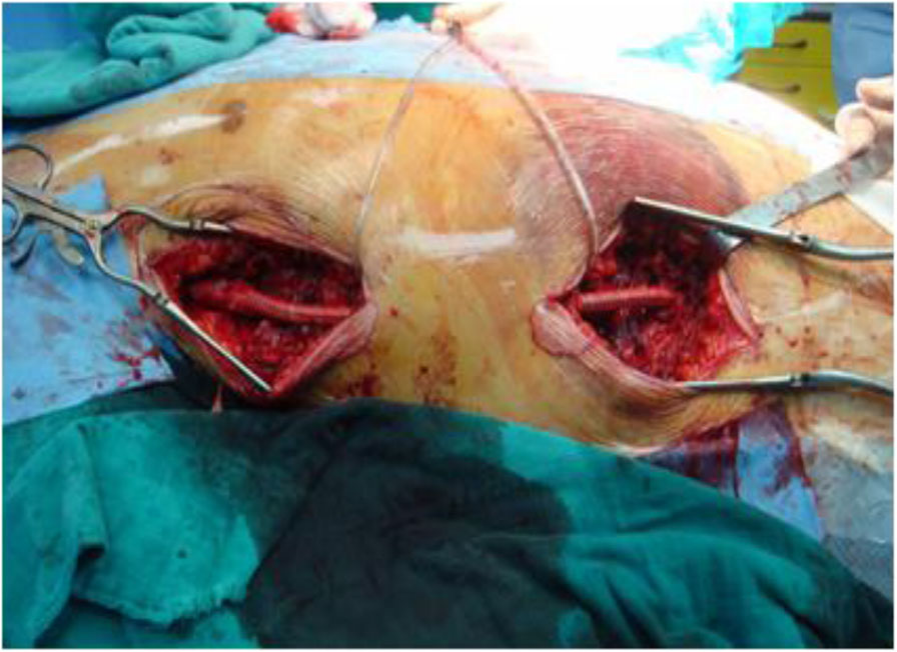
Intraoperative view after the surgical repair with grafts

During the current admission, we found two masses along the side of the AFBG of about 6 cm and 4 cm each. On examination, these masses were painful and tender. The first mass was located at the level of the nipple line, while the second was located at the level of the umbilical line. The overlying skin was tense and blanched. Doppler ultrasonography demonstrated that his left femoral pulse and left distal extremity pulses were absent with monophasic flow. The strength and sensation in his left leg were intact. There were no signs of compartment syndrome. There was a left anterolateral midrib fracture deep to the hematoma. CT of his chest with intravenous contrast showed two non-anastomotic PSAs in the trajectory of the AFBG graft (Fig. 1). His hemoglobin level was 10.0 g/dL, prothrombin time was 1.6 seconds, partial thromboplastin time was 29.7 seconds, and platelet count was 160,000/mL. His renal function was normal with a creatinine clearance rate of 70 mg/m^2^. Echocardiography showed a ventricular ejection fraction of 40%. Consequently, our patient underwent urgent operative repair. The surgical treatment involved flattening the PSAs and the interposition of a prosthetic segment (Figs. 2 and 3). On completion of the procedure, his left femoral pulse was palpable and pedal pulses were present on Doppler ultrasonography. Our patient recovered uneventfully and was well 3 years after discharge.

## Discussion

An AFBG is a routine surgical technique used to revascularize the lower limb. It can be done alone or in conjunction with a femoro-femoral bypass. One of the most interesting benefits of this technique is that it avoids aortic clamping and is associated with low rates of morbidity and mortality when compared with traditional aortobifemoral graft surgery. It also has the advantage that it does not necessarily require general anesthesia [1]. AFBG is therefore a particularly attractive technique for patients with cardiovascular comorbidities.

Despite its benefits, authors have reported lower patency rates for AFBG, estimated to be around 35–71% at 5 years [7, 8]. This percentage can be improved when using rings for external support [8, 9] and can reach a patency rate of 85% [9]. Commonly reported complications of this technique are thrombosis, infection, and PSA [2–15]. This latter complication can occur in a variety of locations, the most frequent being at a femoral artery graft anastomoses, which was where the PSAs were localized in our case. Current studies have theorized that this is due to turbulent blood flow that progressively weakens the arterial wall of end-to-side anastomoses, promoting the development of anastomotic leaks [10]. PSA occurs in 0.8–2.2% of revascularization procedures [11, 12].

Non-anastomotic PSA is an extremely rare complication. We found few cases reported in our literature review, and none reported more than one localization, which makes our report unusual. The first case of post-traumatic non-anastomotic PSA was described by Buche *et al.* in 1992 [15]; they reported the case of a 38-year-old male patient with a PSA secondary to a fall 10 months after his axillofemoral bypass. Piazza *et al.* [4] and Kruger* et al. *[16] each described a case of non-anastomotic PSA, occurring 12 and 29 months respectively after the AFBG. One case was due to a trauma with a seat belt in a cart accident. Etiologies other than traumatism have also been described; iatrogenic disruption as the cause has been reported by several authors [4, 17, 18]. In our case, the non-anastomotic PSA was secondary to a cart accident.

Ultrasonography is considered a reliable tool to diagnose non-anastomotic PSA; however, its main flaw is that it is an operator-dependent procedure [6]. CT angiography is associated with better sensitivity and specificity rates. It allows more precise diagnosis of PSA, in addition to the diagnosis of collections and signs of inflammation or abscess [6]. Intravascular ultrasonography is also crucial in the diagnosis of PSA because it permits proper vessel sizing and an estimation of the severity of pathology [6]. In our case, ultrasonography was complemented by a CT angiogram that permitted an accrual diagnosis.

With regards the treatment and management of this complication, surgical open repair has had excellent outcomes in the first reported cases of non-anastomotic PSA [5–15], and it is still widely used. More recently, minimally invasive techniques have been described in the repair of non-anastomotic PSA. Grochow *et al. *were the first to describe this technique in 2008 [17]. Since then, other cases have been described [18]. The most recently reported case concerned an 82-year-old female patient who presented with a non-anastomotic PSA 15 years after her AFBG; in this case the traumatic PSA was managed via an endovascular approach [6]. In our case, we used an open surgery approach because of the large size of the hematoma, which would not have been easily managed with an endoscopic technique. We were able to repair both of the PSAs with excellent outcomes; our patient remains well 3 years after surgery.

Routine follow-up of these grafts after placement is recommended by most authors, even after a successful repair. Our patient is still attending follow-up appointments.

## Conclusions

A non-anastomotic PSA is a rarely described complication of AFBG; traumatism is the most common etiology. With the advent of new imaging methods, diagnosis of PSA has become easier. Surgery is the standard of care for this entity; however, minimally invasive techniques are being widely used as an alternative to open repair.

## Abbreviations

AFBG: Axillofemoral bypass graft
CT: Computed tomography
PSA: Pseudoaneurysm
